# Purchase behavior in COVID-19: A cross study in Mexico, Colombia, and Ecuador

**DOI:** 10.1016/j.heliyon.2021.e06468

**Published:** 2021-03-25

**Authors:** Emigdio Larios-Gómez, Laura Fischer, Mónica Peñalosa, Mayra Ortega-Vivanco

**Affiliations:** a Benemérita Universidad Autónoma de Puebla (BUAP), México; b Universidad Nacional Autónoma de México (UNAM), México; c Universidad Jorge Tadeo Bogotá, Colombia; d Universidad Técnica Particular de Loja, Ecuador

**Keywords:** Purchase intention in times of pandemic, Purchase behavior intercultural study México Colombia Ecuador, Consumption factors, Commercialization, Consumer attitude, Business decision analysis

## Abstract

This article explores the critical factors of consumption in Mexico, Ecuador, and Colombia, due to confinement and social distancing. Besides, which are the factors that influence the purchase decision. In the proposed model, we tested from quantitative research with a sample of 2,065 online consumers. We analyzed the following statistics: CFA, structural equations, invariance of measurement instruments, and multi-group analysis with the Smart Pls 3 and EQS 6.3 software. The study reveals that time, space, and place in the consumption process is more visible in the purchasing behavior with social distancing, healthy distance, and the commercial restriction caused by the health contingency. In addition to being a health and humanitarian crisis, the pandemic has severe economic consequences worldwide as 1) the increase in unemployment rates, 2) collapsed health systems, 3) education models overwhelmed by technology, 4) supply chains interrupted by the closure of borders, 5) international and domestic tourism suspended due to a lack of sanitary protocols,6) social coexistence curtailed by significantly increased infections and 7) a decreasing demand by consumers for the closure of companies. Despite being Latin American countries, cultural differences were not the priority of consumption in the crisis period due to Covid-19. They significantly change purchasing behaviors, and all have adapted to online and home delivery purchases by the social factor, local consumption, and consumers' attitude. The article presents several considerations on the main factors of consumption in Covid-19 in collectivist countries (North American and South America) such as Mexico, Colombia, and Ecuador and finds no substantial differences with consumers. There are practical implications for companies to adopt online channels and to create sales strategies in the face of the endemic pandemic.

## 1 Introduction

The purpose of this study is to explore the critical factors of consumption in Mexico, Ecuador, and Colombia and the shopping habits due to confinement and social distancing. Besides, what are factors that influence the purchase decisión. The question is, in a pandemic scenario, are there differences in consumption behaviors between Mexico, Colombia, and Ecuador? Are there differences in consumption behaviors between men and women in the generational cohorts of Mexico, Colombia, and Ecuador? Consumers develop habits over time about what to consume (when and where), with highly predictable behaviors. They have various predictive models and consumer knowledge based on repetitive purchasing behavior at the individual and collective level, ranging from the cognitive process in the search for information, decision making in the purchase, and sustainability of waste after consumption.

Although consumption develops habitually and contextually, recent studies on consumer behaviors describe that purchasing habits change from different contexts (Jagdish, 2020a, Jagdish, 2020b): 1) One of them is the social context: It covers the stages in the consumer's life cycle (singleness, marriage, having children, and being a grandfather), including the workplace, the community, neighbors, and friends, 2) The technological context is the most advanced with the emergence of innovative technologies, which have broken old habits in consumption in almost all human aspects, with artificial intelligence (virtual and augmented reality), smartphones, Internet 4.0, and electronic commerce—directly influencing the online buying and consuming process, in new ways of buying products (goods) and consumer services, ideas, and experiences, 3) The context of coexistence, awareness, and well-being, impacting consumption habits in public and shared spaces, as well as respect for the environment and the consumption of healthy products, all with rules and regulations mostly related to public policies and of social behavior that encourage the consumption of socially acceptable, friendly and inclusive products and services and, 4) the external (less predictable) context made up of events that man does not control in one part, such as natural disasters and those that in a certain way he causes, such as global pandemics -including the Covid-19 pandemic-regional conflicts, civil and global wars. These are historical events that have significantly changed both consumption and production and the supply chain worldwide.

This study examines the impact caused in the purchase and consumer behavior in Mexico, Ecuador, and Colombia due to the situation of isolation caused by the COVID 19, based on the theory of favorable decision (Habel et al., 2020), proposes the justification that consumers buy given the perception in economic, social, psychological and cultural factors. Social networks, marketing research networks, and student contacts in the mentioned countries were used, with a reach of 1,007 people from Mexico, 658 from Colombia, and 400 from Ecuador (total of 2,065 people). We match these data with the total population number, the number of infections and deaths from coronavirus from January to July 2020, and with the cultural dimensions of each country (in North America, Mexico with 129,029,171 inhabitants and 395,489 cases of infection with 44,022 deaths, the 11.13% in its case fatality rate; and South America Colombia with 50,921,453 inhabitants and 257,101 cases of infection with 8,777 deaths, 3.4% in its case fatality rate; Ecuador with 17,661,500 inhabitants and 71,181 cases of infection with 2,647 deaths, 3.71% in its case fatality rate).

Thus, the article presents a literature review on the national culture and the main antecedents of consumer purchasing intentions. The methodology then presents the empirical research model, the measurement scales, the sample description, and its procedure. The results present the main research findings and, finally, the conclusions also have implications for academia and practice. The research includes collectivist countries from North American and South America, such as Mexico, Colombia, and Ecuador (Hofstede, 2020a, Hofstede, 2020b). The research model compares these countries and provides results that contribute to the literature on consumer behavior in intercultural and crisis contexts due to academic interest in testing and building new theories in countries with economies in developed.

The results of this lay the foundation for contributions to academic research and business practice. Verma and Gustafsson (2020) describe in an investigation on the emerging research trends of COVID-19 of the field of business and management from a bibliometric analysis approach. That the synopsis of the new related research subareas With COVID-19 and business management is increasing exponentially, indicating that the virus has impacted our present and future lives on several fronts. Core themes include COVID-19's impacts on the economy, value chain, supply chain management, innovation, the service industry, and employment. However, it does not mention research related to purchasing or consumption behavior; the research assumes that in a short period (four and a half months), 107 unique documents were published in Scopus and WoS from 71 different magazines, 272 different institutes, and 61 different countries.

For academic research, this study is among the first to empirically examine the effects of the coronavirus pandemic on consumer purchases in three culturally matched Latin American countries, as evidenced by indices of the six cultural dimensions for selected markets, according to Hofstede, 2020a, Hofstede, 2020b. Also, they are the countries with the highest infection rates and fatality in their population by Covid-19. For business practice (Pantano et al., 2020), the future of commercial (Obal y Gao, 2020). Results provide concrete guidance to companies on sales opportunities to focus on during the coronavirus pandemic.

## 2 Conceptualization of purchase behavior in times of pandemic

Purchase, consumption, and consumer behavior are subject to time, space, and place. In a new context, women work and increasingly have administrative and managerial positions in companies, in addition to the fact that in the family, both spouses work and have fewer children or have changed their desire to be parents for pets. Likewise, the dizzying pace of society's development, the increase in disposable consumption, and practicality have resulted in consumers ordering online, products deliver at home, and even some of the services are already at home.

The time, space, and place in the consumption process is visible now in the purchasing behavior with the social distancing, the healthy distance, and the commercial restriction caused by the Covid-19 health contingency. Despite being, above all, health -and also a humanitarian-crisis, the Covid-19 pandemic presents severe economic consequences throughout the world (Cox, 2020), as an increase in unemployment rates (Hall, 2020; Rushe and Aratani, 2020) collapsed health systems (Washingtonpost, 2020), education models overwhelmed by technology (worldbank, 2020), supply chains disrupted by border closings (Buchholz, 2020; Salcedo et al., 2020), international and domestic tourism suspended due to a lack of sanitary protocols (OECD, 2020), social coexistence curtailed by significantly increased infections (Dubey et al., 2020) and a decreasing demand by consumers for the closure of companies (Szymkowski et al., 2020), mainly in small and medium-sized companies (SMEs). These factors are also part of the purchasing behavior that is changing (Dooley et al., 2010; Foxall, 1979; Lamming, 2000; Habel et al., 2020). To date, there are very few academic works that have examined how purchasing behavior changes during a global pandemic. Sheth (2020c), in one of his recent publications, summarizes the immediate effects on purchasing and consumer behavior due to the Covid-19 pandemic. Table 1 describes the facts on consumer behavior and its modifications in the purchase:

• Hoarding of essential products for daily consumption that result in temporary shortages and shortages. That sometimes hoarding is for safety and protection so as not to expose to the virus and sometimes to find a lucrative business space;
• Improvisation when there are restrictions, existing habits are discarded, and new forms of consumption. The closure and restriction to human conglomerations, the spiritual need, and resilience to Covid-19 have generated the adaptation of virtual meetings in churches, funerals, social events (weddings, concerts, intimate meetings);
• Accumulated demand due to a change in demand in the future for non-essential products. The accumulated or repressed demand, because the markets for goods and especially for entertainment services are closed, will be reflected in a delayed purchase that consumers will make in the first moment of social freedom in the face of the pandemic;
• Use of digital technology not only for the acquisition of products of massive-scale online but also to maintain social, labor, and even to maintain health and follow-up of medical treatments;
• Home deliveries because consumers cannot go to the grocery store or shopping malls, there is the virtual store from home and also intangibles such as services, ideas, and experiences (work, education, healthcare, religion, politics, and entertainment) reversing the flow of buying and consuming;
• Adjustments to the working day have caused them to lose the limits between personal life and working life with extended hours and immediate availability (7/24 in some cases), with days with more than 10 h in front of the computer. Consumers are prisoners in their homes with limited space and too many online activities like working, learning, shopping, and socializing;
• School meetings, with friends and family to share stories and experiences through digital platforms that were beginning to be used for education (and which is now one of the pillars for the transmission of knowledge in times of pandemic) and through social networks, developing faster and more universal adoption of new technologies accelerated by the Covid-19 pandemic; and
• Discovery of the talent for having a more flexible time at home, consumers experience cooking, music, art, and entertainment (the prosumer effect). While reproducing, sharing, producing, and purchasing knowledge (learning) online more creatively.

**Table 1:** Purchase and consumption behaviors due to Covid-19.

Immediate Effects of Purchase and Consumption	Facts on Consumer Behavior
Hoarding Hoarding is a reaction to managing uncertainty and fear of the future supply of products for basic needs. Hoarding is a common practice in war situations, hyperinflation, and the black market. In addition to hoarding, the gray market is also emerging where unauthorized intermediaries hoard the product and raise prices.	Hygienic panic purchases in European countries such as Italy, Spain, and Germany, are repeated in the rest of the world's countries, including the Americas' region. The same was valid for bread, water, meat, disinfection, and cleaning (essential) products. Unleashing food shortages in some regions and, above all, this phenomenon spread to personal protective equipment, in addition to facing counterfeit or non-certified products for the opportunity of compulsive consumer demand.
Improvisation Improvisation to manage the shortage of products or services is an innovative practice. It leads to an alternative to consumption that can become improvisational systemic consumption behavior in crises (civil wars, natural disasters, authoritarian regimes, and pandemics).	Before the Covid-19 pandemic, society reduced recycling, reusing, and recovering waste discarded for convenience and practicality. In this crisis period, weddings, funeral services, and religious celebrations realize through teleconference platforms. Besides scientific and academic meetings, concerts, and school through these platforms (such as Cisco EndPoint, Zoom, Microsoft Team, o Google Meet), we are replacing this traditional location and presence focused events with video conferences asynchronous and synchronous remote interaction.
Accumulated demand In times of crisis and uncertainty, the general trend is to postpone the purchase and consumption of non-essential products or services. The main consequences of denying leisure and recreation services are the pandemic period's effects since superfluous products or goods find a way to be marketed.	The limitations of attending concerts, sports, bars, and restaurants and purchasing luxury products such as cars, jewelry, electronic devices, and even real estate. In addition to going for a walk through the streets, parks, or on the beach. Consumers, at the first opportunity in freedom of purchase, to purchase goods and services wildly, which they did not need but which satisfy the need to buy by buying after the sanitary closure just as it happened in Italy, France, Spain, and Germany, crowded beaches and shops like Zara.
Embracing digital technology Consumers have adopted new technologies and their applications. They have drastically changed the nature and scope of word-of-mouth advice and recommendations, and information exchange in a highly compressed cycle such as the pandemic.	Facebook, YouTube, and WhatsApp, each with more than a billion subscribers and users, are the new platforms for communication, education, work, entertainment, health care, religion, and social interaction. Besides, WeChat, LinkedIn, TikTok, Instagram, Twitter, and WhatsApp have increased. In addition to the arrival and permanent positioning of streaming platforms such as Disney, Netflix, Amazon Prime, ClaroVideo, HBO, and Disney channel.
Adjustments to the working day In The pandemic's face and its consequences, the boundaries between work and home, and between tasks, chatting, and time spent in family life have blurred. It is necessary to respect work schedules and align them to the children's school activities, as they used to be before the pandemic, but now with technological mediation to make the home more efficient and effective.	The provision of work and family times by employers has led consumers to increase their purchases of products at home, electronic payment with debit and credit cards, the consumption of prepared healthy food or not. They are provoking a change in consumer behaviors in schedules, days, quantities, and availability of brands, products, or services.
School meetings with friends and family It seems that school reunions (from primary education to university) and family weddings are now regular and scheduled gatherings under the same approach to share information and experiences from a remote link.	Elementary schools and even the university have graduated their students through virtual ceremonies and droid robots that have replaced the student's presence to receive their studies' certificate, but not the emotion and experience of living it directly.
Discovery of talent Consumers are becoming producers of their products or services. In other words, in the pandemic's face, the prosumer they carry within, and even the entrepreneur has emerged, given the health circumstances and market access. They are discovering the potential for innovation and commercial success.	YouTube increased the registration of channels by entrepreneurs of all ages, who either by losing their job or spending time at home due to the pandemic, have entered the networks to teach, share, commercialize or exchange goods and services made by themselves. Do-it-yourself channels in cooking, repairs, art, languages, academic knowledge, tourism, wellness, and health have increased.

The framework proposed for this study examines and contrasts vital factors related to the purchasing decision-making process and its consequences. These elements operationalize as antecedent variables (generational, economic, social, psychological, and cultural cohorts) that can influence purchase intention and behavior in a crisis economy. Based on a review of the literature, purchase intention often select as the basis for the study of purchasing behavior. The literature shows that purchase intention is the main predictor of any consumption behavior (Fishbein and Ajzen, 1977); therefore, this work treats purchasing behavior as consumer behavior. The main theories that have impacted human behavior prediction from social psychology and culture determine purchase and consumption factors.

According to Verdugo and Ponce (2020), few intercultural studies on consumer behavior, including Latin American countries. In addition to the literature, several studies are related to western versus eastern countries and comparisons between individualist versus collectivist countries, with countries in North America, Western Europe, and Asia (Mazaheri et al., 2011; Engelen and Brettel, 2011; Peña-García et al., 2020).

**Figure 1:**
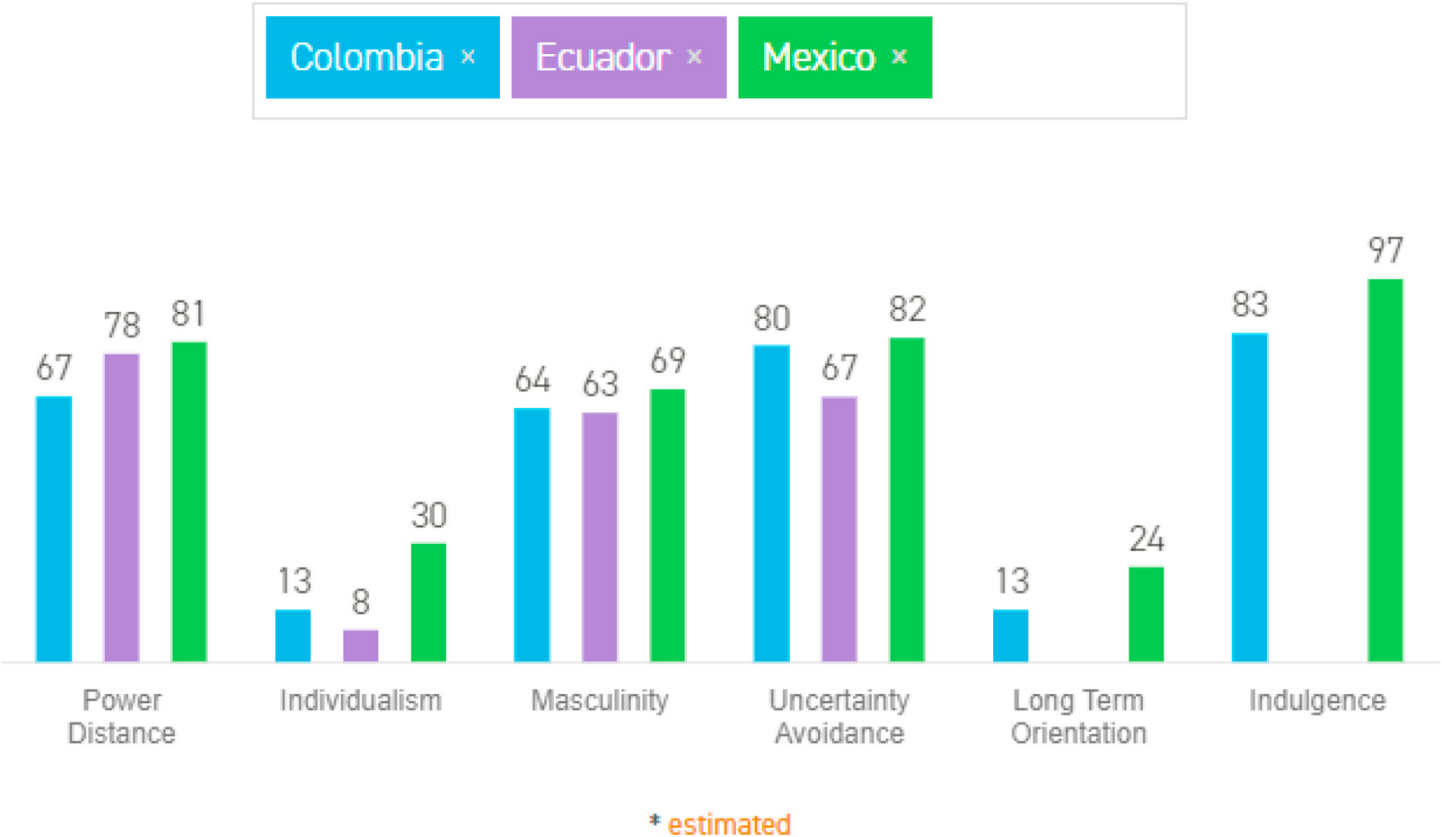
North American country (as Mexico) Vs. South American countries (as Colombia and Ecuador). Source: (Hofstede, 2020a, Hofstede, 2020b).

Therefore, the present investigation examines North American countries such as Mexico with two countries in South America, such as Colombia and Ecuador. We can say that Mexico (score 30) is less collectivistic than Colombia (score 13) or Ecuador (score 8). However, it still is collectivistic because “…people belong to ‘in groups’ that take care of them in exchange for loyalty…” (Hofstede, 2020a, Hofstede, 2020b). We got this reflexión about country comparison in the 6-D Model© to showing the values for the six dimensions of the cultural survey in the Culture Compass™ Figure 1:

Mexico, with a score of 30, is considered a collectivistic society. The manifests in a close long-term commitment to the member ‘group': a family, extended family, or extended relationships. Loyalty in a collectivist culture is paramount and over-rides most other societal rules and regulations. Society fosters strong relationships where everyone takes responsibility for fellow members of their group. In collectivist societies, offense leads to shame and loss of face, employer/employee relationships perceive in moral terms (like a family link), hiring and promotion decisions take account of the employee's in-group, management is the management of groups.

At a score of 13, Colombia is amongst the lowest individualist scores. In other words, it lies amongst the most collectivistic cultures globally, beaten only by Ecuador, Panama, and Guatemala.

Since the Colombians are highly collectivistic people, belonging to an in-group and aligning with their opinions is very important. Combined with the high PDI scores, groups often have their strong identities tied to class distinctions. Loyalty to such groups is paramount, and often it is through “corporative” groups that people obtain privileges and benefits that they do not have in other cultures.

At a score of 8, Ecuador is amongst the lowest individualist scores. In other words, it lies amongst the most collectivistic cultures in the world, beaten only by Guatemala. Since the Ecuadorians are highly collectivistic people, belonging to an in-group is very important. Combined with the high PDI scores, groups often have strong identities tied to race and class distinctions. Struggles for power among different political factions, though frequent, seldom have become very violent.

The model describes the comparative between Mexico, Colombia, and Ecuador and provides results that contribute to consumer behavior literature in intercultural contexts. Besides, and provide relevant answers nationally and internationally due to academic interest in testing and building new theories in countries underdeveloped economies.

Hofstede (2001) points out that culture can, therefore, be a set of artifacts, models, and patterns of behavior through which a society expresses itself and reproduces itself. Its broadest sense includes practices, codes, rituals, norms, rules, and customs. The cultural dimension occurs in three types of mental programming levels: individual, collective, and universal. The people's personality defines the first, and therefore, a part of it is inherited. Another they learned, explaining why people with different personalities have the same conditions and social environment. Second, the collective level is the one that determines the culture and therefore is the mental programming fully learned. Moreover, the third level is universal, which is entirely inherited and is explained by the nature of being human. So, the cultural dimension can measure concerning other cultures, initially identifying four basic dimensions of national culture in 1980 distance from power, aversion to uncertainty, individualism, and masculinity" and recently Hofstede et al. (2010, 2015) adds Uncertain Dislike, Long-Term Orientation, and Indulgence.

Thus, culture is not so much a property of individuals or groups, but rather a tool to understand and learn the differences commonly attributed to national culture as a critical element from which consumer behavior can differ in areas (Burton, 2008; Kumar and Pansari, 2016). In this research, the national culture is a critical variable in differentiating consumer behavior in Covid-19 times from three nations with Latin American cultures (Mexico, Colombia, and Ecuador) that have lagged behind these intercultural studies in behavior. Of the consumer and help fill an essential gap in the scientific literature on Latin American culture (See-Pui, 2013; Verdugo and Ponce, 2020; Wanick et al., 2019). Table 2 shows the indices of the six cultural dimensions for the selected markets, according to Hofstede, 2020a, Hofstede, 2020b.

**Table 2:** The 6 dimensions of Hofstede: Mexico Vs. Colombia/Mexico Vs. Ecuador.

Dimension	Mexico	Colombia	Δ	Ecuador	Δ
Distance power	81	67	14	78	3
Individualism	30	13	17	8	22
Masculinity	69	64	5	63	6
Uncertain dislike	82	80	2	67	15
Long-term orientation	24	13	7	-	-
Indulgence	97	83	14	-	-
(Δ) Delta=<30.					

**Figure 2:**
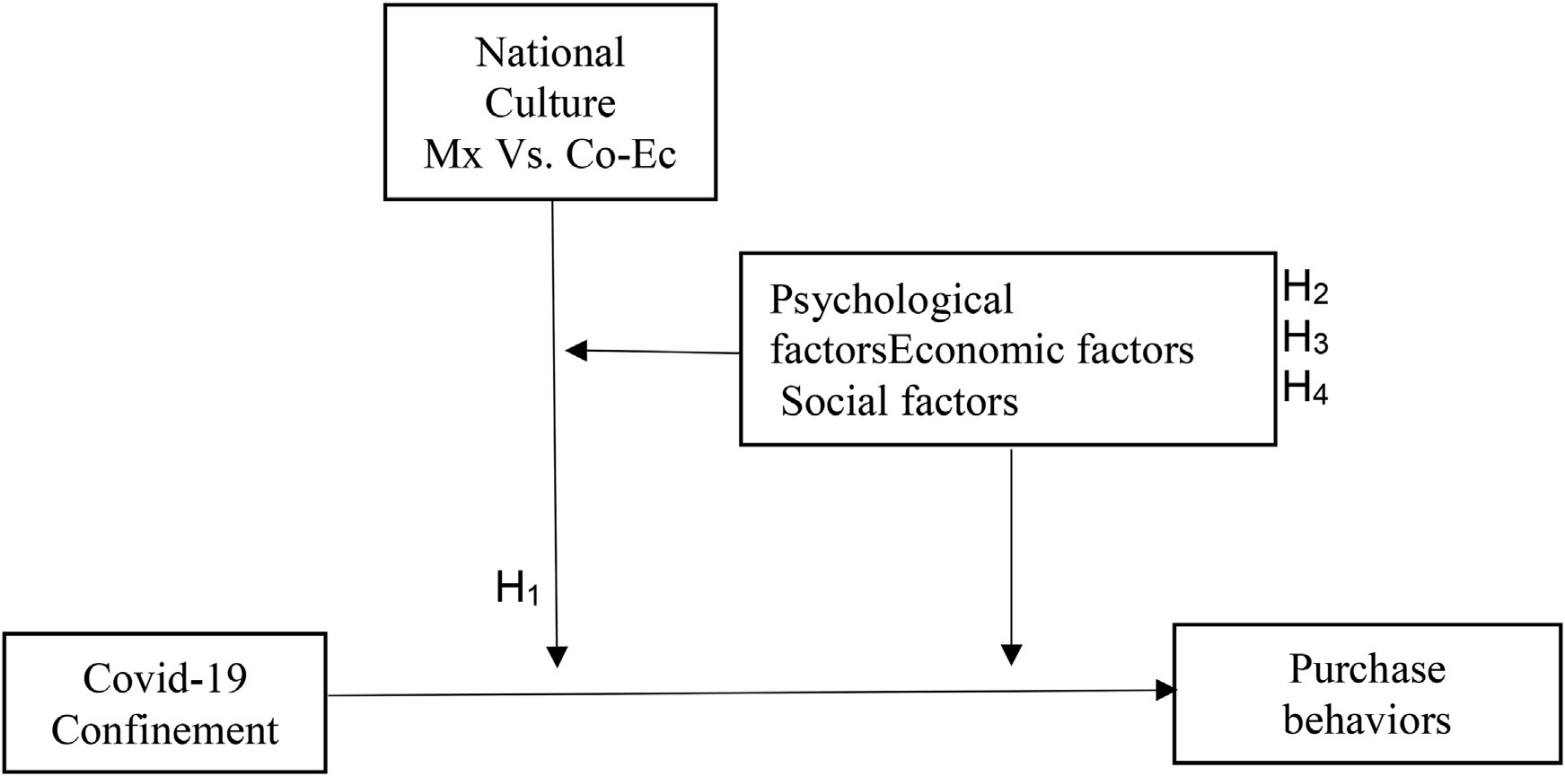
Hypothetical Model. Source: Own elaboration.

Now, according to the bipolar dimensions of Inglehart (1997), Mexico, Colombia, and Ecuador do not differ significantly in the dimension that includes traditional values versus secular/rational values. Figure 2 shows the countries' location, according to the results of the Wave 6 World Values Survey (WVS) (Inglehart et al., 2014). This dimension analyzes the process of change from traditional to modern societies. As can be seen, although Mexico (located in North America), compared to Colombia and Ecuador (located in South America), for the three countries, their societies are traditionalists. Where religion, family ties, gender roles, and national pride are essential. Based on the comparative, Mexico, Colombia, and Ecuador are at the pole of traditional values; therefore, based on this basis, the hypothetical model is proposed in Figure 2.

WVS Wave 6 survey of 2014, before the global crisis caused by Covid-19, the results do not reflect reality before the pandemic. "The pandemic crown shames and isolates us, making us lonely individuals and isolated countries, hiding, scared of each other, dying without saying goodbye." (Hofstede, 2020a, Hofstede, 2020b, s/p). In a scenario before Covid-19, one would not expect differences between national cultures in consumer behavior in Covid-19 times. Nevertheless, in a pandemic scenario, are there differences in consumption behaviors between Mexico and Colombia, and Ecuador?

Examining purchasing behaviors can use for the implementation of a new distribution channel. Moreover, it helps managers to determine and decides which geographic and virtual markets with the segments actual consumer behavior (Morwitz et al., 2007); therefore, your study is of utmost importance to the success of any retailer in geographic distribution channels and now online. From the pre-purchase stage, it is necessary to know the motivational aspects that affect customer behavior to predict consumer behavior, knowing the attitudes, evaluations, and internal and external factors that ultimately generate the purchase (Armitage and Conner, 2001; Fishbein and Ajzen, 1977). Furthermore, purchasing behavior has a different perspective of various marketing aspects, in addition to traditional purchases in physical stores, such as green marketing, luxury brands and products, B2B transactions, and, finally, online shopping (Beuckels and Hudders, 2016; Nguyen et al., 2016; Wei and Ho, 2019; Sundström et al., 2019). However, buying and consuming had not several studies from crisis perspectives (Covid-19). For the present study, the origin and residence in the study subjects' countries will be taken as culture: Mexico, Colombia, and Ecuador.

Is there a difference in purchasing behaviors due to social confinement due to the Covid-19 health contingency impact between Mexico, Colombia, and Ecuador?

H1 There is a difference in purchasing behaviors between Mexico, Colombia, and Ecuador.

On the other hand, attitudes are learned and developed throughout the consumer's life and are difficult to change. However, they can influence the psychological motivation's satisfaction that causes a change as new concepts about the idea or object consumed (Lien and Cao, 2014; Shaouf et al., 2016). Attitude determines an individual's positive or negative predisposition to the behavior (Allport Attitudes and Murchinson, 1935; Fishbein and Ajzen, 1977). Recent studies, such as the one carried out by Torales et al. (2020), during the confinement of the pandemic, report that the population is having psychological reactions and states related to attitude and motivation in mental health and physical. In this research, attitude and motivation as a psychological factor is understood as (a): Mental Attitude; (b): Health and Exercise and (c): Prediction or Attitude towards buying and consuming behavior in Covid-19 times that must be influenced by social confinement.

So, which is the psychological factor that difference in purchasing behaviors due to social confinement due to the Covid-19 health contingency impact between Mexico, Colombia, and Ecuador?.

H2 The difference in purchasing behaviors between Mexico, Colombia, and Ecuador is due to the attitude of consumers.

Planned Behavior Theory (TPB) is defined as "an extension of reasoned action theory" by adding perceived behavioral control to external factors that can influence a person's behavior, emphasizing the perception of behavioral control as of "greater psychological interest" (Ajzen, 1991, p. 181–183). However, purchasing behavior occurs in a specific context and varies according to external conditions (Groening et al., 2018). Therefore, behavior depends on external conditions, that is, "all external sources of support or opposition to behavior, whether physical, financial, legal or social" (Guagnano et al., 1995, p. 702; Stern et al., 1999).

For this research, external economic conditions refer to the consumer's behavior to buy local products (local production and consumption of local or regional products) and reduce unnecessary products as a psychological factor of purchase. So, it is understood as (a): National Production and (b): Consumption of local products towards purchase and consumption in times of Covid-19 that must be influenced by social confinement.

So, the economic factor (local or regional) that difference in purchasing behaviors due to social confinement due to the Covid-19 health contingency impact between Mexico, Colombia, and Ecuador?.

H3 The difference in purchasing behaviors between Mexico, Colombia, and Ecuador is local consumption.

The buyer's characteristics could be personal, psychological, cultural, and social. These characteristics affect the purchase decision process: the need for recognition, information search, evaluation of substitutions, purchase decision, and post-purchase. The social factors that influence consumer behavior are the reference groups, family and social roles and states, for example, friendship groups, family groups, shopping groups, working groups, virtual groups or communities, and consumer action (Schiffman and Kanuk, 2001; Kotler and Armstrong, 2008; Solomon, 2009; Cornelis, 2010.) According to the Engel-Kollat-Blackwell (EKB) model, are consumer resources, motivation, participation, knowledge, attitudes, personality, values, and lifestyle (Engel et al., 1995). For the present study, the factors influencing behavior consumption are social, personal, and family topics (such as the cultural, economic, and demographic, explained previously) and individual. Based on this, friendship groups, family groups, shopping groups, working groups, virtual groups or communities, and consumer action describe the social factors: (a) personal likes and (b) social networks (with family and Friends) in the behavior of buying and consuming in Covid-19 pandemic period. So, which is the social factor that difference in purchasing behaviors due to social confinement due to the Covid-19 health contingency impact between Mexico, Colombia, and Ecuador?.

H4 The difference in purchasing behaviors between Mexico, Colombia, and Ecuador is due to social networks (with family and Friends).

Based on the literature review, a research model to compare the relationships (Figure 2).

## 3 Methodology

### 3.1 Research context

To examine the impact caused by Covid-19 on the purchase and consumer behavior in Mexico, Ecuador, and Colombia, we contacted to study survey through social networks (Facebook, Twitter, WhatsApp). Furthermore, just the Baby Boomer generation from 56 to 65 years old, we contacted by telephone), by personal and institutional email at the members of marketing research networks (International Network of Marketing Researchers -RIIM, the Latin American Marketing Network -RLM and the Ecuadorian Marketing Network -REM) and through the contacts of students (in administration, business, and marketing) in the mentioned countries. We collected the information in the period from May 31 to June 30, 2020, ensuring that the population of the three countries was already in confinement asylees with healthy distance policies, at least two months after the World Health Organization (WHO) declared pandemic for SARS-Co2 or Covid-19 New Coronavirus (March 11). Besides taking into account that the three Latin American countries have been affected by the highest infection rates and fatality in their population by Covid-19.

### 3.2 Samples and procedures

To understand the consumption in men and women belonging to Mexico, Ecuador, and Colombia due to the isolation situation caused by COVID 19. The online survey was about 2,068 people over 18 years of age (with a reach of 1,010 people from Mexico, 658 from Colombia, and 400 from Ecuador). Between May and June 2020, in the countries studied. The sample and participation were voluntary and limited to consumers born and raised in selected countries to ensure they were from the national cultures analyzed in this study. After applying the filters, 2,062 valid applications (99.85%) were obtained: 1,007 people from Mexico (49%), 658 from Colombia (32%) and 400 from Ecuador (19%).

Most of the participants are women (61%), single (68%), and higher education with a degree (76%). Ages range from 18 to 25 years for 36% and between 26 to 43 years for 25%. We used IBM SPSS Statistic 25 and Smart-PLS 3.3.2 programs to process the data; Table 3 shows demographic information of the sample by national culture.

**Table 3:** Demographic information of the samples.

Variable	ítems	Mexico		Colombia		Ecuador	
		Frequency	%	Frequency	%	Frequency	%
Gender	Women	642	64%	359	55%	254	64%
	Man	365	36%	299	46%	146	37%
Age	from 18 to 25 years	403	40%	236	36%	102	26%
	from 26 to 43 years	199	20%	225	34%	95	24%
	from 44 to 55 years	176	17%	104	16%	149	37%
	from 56 to 65 years	229	23%	93	14%	54	14%
Marital status	Married	312	31%	211	32%	141	35%
	Singles	695	69%	444	68%	259	65%
Education	Basic	9	1%	13	2%	19	5%
	Average Superior	171	17%	164	25%	113	28%
	Superior	827	82%	481	73%	268	67%

### 3.3 Variables and measures

For the construct, consumption behavior in men and women in Mexico, Ecuador, and Colombia due to the situation of isolation caused by COVID 19 based on the theory of favorable decision (Habel et al., 2020), an instrument was designed for research with measurement scales previously validated in the literature. It was validated qualitatively with experts in consumer behavior, who contributed theories and frontier literature to adjust elements that integrated the study constructs. The measures for cultural factors (national culture) were adapted from Hofstede (2015), from the studies by Groening et al. (2018), the scales were adapted to measure social factors. We adapted the scale to measure psychological factors from the studies by Lien and Cao (2014), Shaouf et al. (2016) and de Torales et al. (2020), Torales, O'Higgins, Castaldelli-Maia, and Ventriglio (2020). The study by Sheth (2020c) let adapted for the scale of purchasing behaviors in the time of Covid-19 and the studies of Schewe and Meredith (2004), Schlossberg (2016), Bernstein (2015). Table 4 shows the adapted scales.

**Table 4:** Adapted scales.

Construct	Factors (variables)	Ítems	Scale
	Cultural factors	CN0_Cultura Nacional (Country)	Hofstede, 2020a, Hofstede, 2020b
	Generational cohorts	PS1_Gender PS2_Cohorte Generational PS3_Civil Status PS4_Education	Schewe and Meredith (2004); Schlossberg (2016); Bernstein (2015)
Positive decision theory (Habel et al., 2020)	Psychological factors (Attitude)	FP1.1_Humor before the Covid-19 FP1.2_Family Communication	Lien and Cao (2014) Shaouf et al. (2016) Torales, O'Higgins, Castaldelli-Maia and Ventriglio (2020)
	Psychological factors (Health)	FP2.1_Exercise and Health FP2.2_Food changes	
	Psychological factors (Prediction)	FP3.1_Optimism FP3.2_Buy Insurance	
	Social factors (Communication with others)	FS1.1_Use of Social Networks FS1.2_Network Professional FS1.3_Using WhatsApp FS1.4_VideoconferenciaRecreativa FS1.5_VideoconferenciaProfesional FS1.6_Listen Radio (removed)	Groening et al. (2018)
	Social factors (Personal)	FS2.1_Streaming movies FS2.2_Streaming music and games FS2.3_Interest Kitchen	
	Economic factors (Sustainability)	FE1.1_Local Production FE1.2_Home Consumption	
	Economic factors (Basic consumption)	FE2.1_Reduction of plastics FE2.2_Changes Changes FE2.3_Environment (removed)	
	Purchase behaviors	CC2_Compras Online CC3_Increase in purchases CC1_Home Delivery	Sheth (2020c)

### 3.4 Reliability and validity

A second-order confirmatory factor análisis (CFA) let performed to assess the scales' reliability and validity using the maximum likelihood method using SMART PLS 3.3.2 statistical software (Ringle et al., 2015). The values of indicators that presented the minimum or exceeded the recommended value of 0.7 in their loads were maintained. We eliminated two indicators: FS1.6_Listen Radio and FE2.3_Environment (eliminated), presenting loads less than 0.6, respectively. Likewise, we preserved the indicators that presented a Cronbach's Alpha greater or equal, which provides evidence of reliability and justifies the scales' internal reliability (Hair et al., 2010; Nunnally ly Bernstein, 1994). However, the composite reliability (CRI) is considered better internal consistency and must be greater than 0.60 (Bagozzi and Yi, 1988); we also preserved the scales that exceeded this measure. The extracted mean-variance index (AVE) was more significant than 0.50 (Fornell and Larcker, 1981) in each one of the scales, so the variance of the construct obtained from its indicators is adequate. The results of the study can see in Table 5.

**Table 5:** Internal consistency and convergent validity of the theoretical model.

Variable	Indicator	Loads >0.70	Value t >1.96	Cronbach's Alpha	CRI >0.70	AVE >.050
Psychological factors (Attitude)	FP1.1_Humor before the Covid-19	0.858∗∗∗	22464	0.726	0.777	0.635
	FP1.2_Family Communication	0.764∗∗	22.546			
Psychological factors (Health)	FP2.1_Exercise and Health	0.718∗∗	12.045	0.705	0.725	0.579
	FP2.2_Food changes	0.928∗∗∗	40.662			
Psychological factors (Prediction)	FP3.1_Optimism	0.769∗∗	6.132	0.743	0.718	0.565
	FP3.2_Buy Insurance	0.757∗∗∗	13.684			
Social factors (Communication with others)	FS1.1_Use of Social Networks	0.774∗∗	42.719	0.789	0.855	0.541
	FS1.2_Network Professional	0.772∗∗	41.046			
	FS1.3_Using WhatsApp	0.745∗∗	41.516			
	FS1.4_VideoconferenciaRecreativa	0.741∗∗	37.958			
	FS1.5_VideoconferenciaProfesional	0.765∗∗	32.693			
	FS1.6_Listen Radio (removed)	0.523				
Social factors (Personal)	FS2.1_Streaming movies	0.703∗∗	22.250	0.758	0.734	0.581
	FS2.2_Streaming music and games	0.731∗∗	26.022			
	FS2,3_Interest Kitchen	0.716∗∗	15.148			
Factores Economic factors (Sustainability)económicos (Sustentabilidad)	FE1.1_Local Production	0.832∗∗∗	27.606	0.711	0.734	0.586
	FE1.2_Home Consumption FS2.3_Environment (removed)	0.764∗∗	12.042			
Economic factors (Basic consumption)	FE2.1_Reduction of plastics	0.722∗∗	2.557	0.708	0.776	0.508
	FE2.2_Changes Changes	0.976∗∗∗	179.627			
	FS2.3_Environment (removed)	0.508				
Purchase behaviors	CC2_Compras Online	0.752∗∗	43.926	0.743	0.803	0.578
	CC3_Increase in purchases	0.848∗∗∗	99.058			
	CC1_Home Delivery	0.752∗∗	14.169			

S-B X2 5095,456 at 2150df; (S-B X2/df) 2.36; p- 0.000; RMSEA 0.065; NFI 0.825; NNFI 0.828; CFI- 0.845 - Parameters restricted to this value in the identification process; Level of significance ∗∗∗ á p < 0.001; ∗∗ á p < 0.05 CRI- Composite Reliability Index; AVE- Average Variation Extracted.

Therefore, we found that the original model showed level adjustment problems; as explained in previous lines, it was necessary to eliminate two observable variables for the entire theoretical model. Their factor loads were below the value of 0, 6 suggested. The model fit showed satisfactory values (EQS 6.3 software): NFI = 0.825; NNFI = 0.828; CFI = 0.845; RMSEA = 0.065; SRMR = 0.051. The model's composite reliability for the total sample we verified with values between 0.727 and 0.855 in all constructions. Furthermore, AVE values were greater than 0.5.

With the goodness of fit of the established model, a CFA was performed for each subsample to determine the reliability and validity of the measurement instrument for the Mexican, Colombian and Ecuadorian subsamples through the PLS Multi-Group Analysis in SmartPLS 3 (Ringle et al., 2015). The results are shown, respectively, in Table 6. Similarly, as in the global sample, the indicator values were kept with a minimum of 0.7 in their loads. We preserved the indicators that presented a higher Cronbach's Alpha and the compound reliability to 0.60. The mean-variance index extracted was more significant than 0.50 in each of the scales. In all the sub-samples, the scales of the construct's reliability and validity obtained from its indicators are adequate.

**Table 6:** Internal consistency and convergent validity of the model adjusted by subsamples.

Variable	CRI >0.70	AVE >.050	CRI >0.70	AVE >.050	CRI >0.70	AVE >.050	CRI >0.70	AVE >.050	CRI >0.70	AVE >.050
	MAN		WOMEN		MEXICO		COLOMBIA		ECUADOR	
FP1	0.800	0.667	0.761	0.615	0.764	0.619	0.794	0.661	0.761	0.615
FP2	0.725	0.586	0.724	0.573	0.781	0.548	0.739	0.594	0.713	0.560
FP3	0.716	0.558	0.711	0.566	0.721	0.518	0.753	0.605	0.727	0.577
FS1	0.856	0.543	0.854	0.539	0.855	0.542	0.859	0.550	0.834	0.503
FS2	0.749	0.504	0.723	0.466	0.716	0.560	0.762	0.526	0.723	0.566
FE1	0.720	0.584	0.730	0.575	0.735	0.582	0.755	0.608	0.751	0.601
FE2	0.784	0.511	0.573	0.506	0.753	0.505	0.632	0.524	0.773	0.500
CC	0.807	0.583	0.802	0.575	0.747	0.501	0.839	0.635	0.780	0.544

The invariance of the measurement instrument consists of introducing restrictions to validate that the latent variables represent the same in the three groups (Aldás, 2013; Hair Jr. et al., 2018). This investigation analyzes the same form and load factor using PLS Multi-Group Analysis in SmartPLS 3 and EQS 6.3 software. As shown in the results, Table 7 shows the results to determine if the constraints can be sustained and confirm the measurement instrument's invariance. Cheung and Rensvold (2002) proposed an approach based on the CFI difference between the results after the same form, factor load, or ΔCFI. This approach indicates that when ΔIFC> 0.01, the applied constraints cannot be sustained, whereas when ΔCFI ≤ 0.01, the constraints are sustained, which means that the latent variables are the same in the subsamples. This study found that ΔCFI = 0.005 (CFI 0.983 - CFI 0.978); consequently, invariance is confirmed (Henseler et al., 2009).

**Table 7:** Invariance test.

Group	X^2^	df	X^2^/df <3	RMSEA <0.05	SRMR P a 0	NFI ≥0.95	CFI ≥0.95
Mexico (n = 1007)	2345.009	1410.58	1.66	0.050	0.084	0.956	0.960
Colombia (n = 658)	1918.863	921.45	2.08	0.046	0.085	0.937	0.977
Ecuador (n = 400)	1090.747	784.00	1.39	0.047	0.087	0.983	0.983
Equal Form	5353.881	2150	2.49	0.046	0.087	0.975	0.983
Equal load factor	5835.730	2258	2.58	0.049	0.087	0.973	0.978

Likewise, it could note that the goodness of fit statistics and reference criteria are adequate. For the absolute fit, having a Chi-square/degrees of freedom (X2/pdf) ratio less than 3, the mean square root of the approximation error (RMSEA) less than 0.05, and the root of the root mean square residual (SRMR) with approaches to zero. In comparative adjustment, the Normalized Adjustment Index (NFI) greater than or equal to 0.95, and also the Comparative Adjustment Index (CFI) is greater than or equal to 0.95.

### 3.5 SEM and multi-group analysis

The H_1_ hypothesis test, through the structural model and MGA, approaches the evaluation using two different non-parametric procedures, bootstrap-based MGA (Henseler et al., 2009) and the permutation test (Chin and Dibbern, 2010). For the MGA permutation method, p < 0.05 indicates a five percent level means a significant difference between two groups, Mexico about Colombia, and Ecuador. Similarly, the permutation test was applied where the p-value is < 0.05 gives significance in the relationship between groups (Chin and Dibbern, 2010; Edgington and Onghena, 2007), as observed in Table 8, we accepted the H_1_: There is a difference in purchasing behaviors between Mexico, Colombia, and Ecuador.

**Table 8:** Mexico Vs. Colombia-Ecuador: Hypothesis 1.

Hypothesis	R2 Differences	R2 permuted	P-value Permutation <0.05	Result
H_1_: Mexico Vs. Colombia-Ecuador	-0.121	-0.006	0.009	Accepted

Once the measurement model's convergent and discriminant validity was assured, the relationships between the variables were measured, beginning by obtaining the different statistical parameters through the bootstrapping method (5000 sub-samples). For Henseler's bootstrap-based MGA method (p < 0.05 or p > 0.95) between two categories (Henseler et al., 2009), a p-value of less than 0.05 or greater than 0.95 indicates significant changes. As shown in Table 9, we rejected the H_2_: The difference in purchasing behaviors between Mexico, Colombia, and Ecuador is due to the attitude of consumers. Furthermore, we accepted the H_3_: The difference in purchasing behaviors between Mexico, Colombia, and Ecuador is due to local consumption is accepted and, and H_4_: The difference in purchasing behaviors between Mexico, Colombia, and Ecuador is due to social networks (with family and Friends).

**Table 9:** Mexico Vs. Colombia-Ecuador: Hypothesis 2,3 y 4.

Hypothesis - Relationships	Path (β)	t-value >1.96	P-value <.05	Result
H_2_: FP-1 MEN - > CC-CONS (psychological factors Mental Attitude)	0.036	1.398	0.163	Rejected
H_3_: FE1-NAC - > CC-CONS (economic factors Consumption of local)	0.059	2.526	0.012	Accepted
H_4_: FS1-COM - > CC-CONS (social factors family and Friends)	0.150	5.957	0.000	Accepted

## 4 Discussion

With the results obtained, we analyzed the data from the perspective of culture, and we could differentiate the consumer behavior in Mexico, Colombia, and Ecuador. A country's culture is a tool to understand and learn the differences commonly attributed to national culture. It is a critical element of consumer behavior in different areas or concepts (Burton, 2008; Kumar and Pansari, 2016). The impact generated by the health contingency caused by Covid-19 on purchasing behaviors due to social confinement significantly influences consumers in Mexico and Colombia. It is different between these three countries (H1: Mexico-Colombia-Ecuador, p = .009). To find differences in purchasing and consumption behavior, in more detail, we proceeded to analyze the behaviors in an AMS, which allows us to identify the specific categories and variables of purchase. According to the bipolar dimensions of Inglehart (1997), Mexico, Colombia, and Ecuador do not differ significantly in the dimension that includes traditional values versus secular/rational values. The Mexican, Colombian, and Ecuadorian consumers' behavior is different in a period of humanitarian, economic, and health crisis caused by the Covid-19. In other words, purchasing behavior is different from one country to another, even though the three countries are traditionalists. Mexico is in North America, compared to Colombia and Ecuador located, are in South America, where religion, family ties, gender roles, and national pride are essential and behave similarly. In the face of a health crisis, the purchasing behavior is not the same.

Based on the comparative, Mexico, Colombia, and Ecuador are at the pole of traditional values. Therefore, based on this topic, the hypothetical model is proposed. It will directly help the business readers of this study to make business decisions in their companies. Besides, these detailed analyzes will provide theory on consumer behavior in a period of a pandemic since these intercultural studies on consumer behavior and help fill an essential gap in the scientific literature on Latin American culture (See-Pui, 2013; Verdugo and Ponce, 2020; Wanick et al., 2019).

About Hypotheses H_2_: The difference in purchasing behaviors between Mexico, Colombia, and Ecuador is due to the attitude of consumers, related to psychological factors, consumption does not present a relationship with any of the categories or variables proposed in the study, reason why the hypothesis we rejected (p = 0.163). A mental attitude such as humor and family life and protection and optimism is, in general, has no relation to consumption or purchasing behavior in the pandemic period. As Lien and Cao (2014) and Shaouf et al. (2016) mentioned, the attitudes are learned and developed throughout the consumer's life and are difficult to change. However, they can be influenced by the psychological motivation's satisfaction that causes a change as new concepts are learned about the idea or object consumed. In the face of a pandemic like Covid-19, the consumer's attitude does not change his or her purchasing behavior. It continues to preserve its purchasing habits, determined by their individual's positive or negative predisposition with the behavior (Allport Attitudes and Murchinson, 1935; Fishbein and Ajzen, 1977).

However, based on recent studies, the psychological factors produced by exercising and changing eating habits are significantly related to Covid-19 influences in the purchase and consumption pandemic period. Torales, O'Higgins, Castaldelli-Maia, and Ventriglio (2020), during the pandemic confinement, report that the population is having psychological reactions and states related to attitude and motivation in mental health and physical.

Hypothesis H_3_:The difference in purchasing behaviors between Mexico, Colombia, and Ecuador is due to local consumption is firmly accepted since it is the only hypothesis that presents p < 0.05 (p = 0.012) in all categories and variables. The difference in purchasing behaviors between Mexico, Colombia, and Ecuador is due to local consumption. Purchasing behavior occurs in a specific context and varies according to external conditions as external sources of support or opposition to behavior. Whether physical, financial, legal, or social as mentioned Guagnano et al. (1995); Stern et al. (1999); and recently Groening et al. (2018), as in this case the pandemic due to Covid-19.). In other words, the impact generated by the health contingency caused by Covid-19 significantly influences consumers when purchasing essential products due to social confinement, both in Mexico, Colombia, and Ecuador. Significantly they stopped buying superfluous products (item FE2.1) and increased the consumption of sustainable products (item FE2.2), increasing (item CC3) the behavior of online shopping (item CC2) and at home (item CC1). Sheth (2020c), in a recent qualitative exploratory study, in one of his publications, summarizes the immediate effects on purchasing and consumer behavior due to the Covid-19 pandemic and describes some facts on consumer behavior and its modifications in the Immediate purchase of the Covid-19 pandemic such as the use of technology (online shopping), home delivery and the purchase of necessities.

The social factors that influence consumer behavior are related to exogenous consumers' deducible influences such as reference groups, roles, and family and social states (Schiffman and Kanuk, 2001; Kotler and Armstrong, 2008; Solomon, 2009; Cornelis, 2010). Moreover, the results corresponding to the social factors represented in hypothesis H_4_ (The difference in purchasing behaviors between Mexico, Colombia, and Ecuador is due to social networks (with family and Friends) related to consumption in the pandemic period. In both men and women of different ages who participated in the survey, we accepted the hypothesis; there is a relating the consumption in Covid-19 with Social Networks (present a significant p-value with p = 0.000). The subjects surveyed present a consumption profile that, witnessing the analog to digital change, aims to have a lifestyle that gives them freedom and flexibility (Schewe and Meredith, 2004), buying analogously and digitally. Consequently, Mexico, Colombia, and Ecuador present a significant relationship between social networks and their purchasing behavior in pandemic periods (for example, friends, family groups, shopping groups, workgroups, virtual groups, or community consumer action).

## 5 Conclusions

Mexico, Colombia, and Ecuador are emerging economies. Most of the companies in these countries are Mipyme (micro, small and medium-sized companies), presenting the same characteristics as few employees, little capital, and an owner who carry out most business activities (marketing, quality, accounting, or purchasing and logistics administration). Most of them are family-owned companies with a short-term vision focused on results and strictly on sales. The most severe problems are the lack of experience in business administration and limited knowledge of the market, which generally leads to the company's dissolution. So they must look ahead, plan, and not just focus on day-to-day sales and marketing. Recently, companies of all sizes, especially MSMEs, have approached educational institutions and scientific research to understand consumer behavior better, both in traditional (face-to-face) and virtual markets. Likewise, the government and institutions play a fundamental role in linking market knowledge with small and medium business owners to acquire strategies and benefit from commercial or business research projects, as is the example in this article.

This research focused on examining the motivational factors that determine consumer behavior in the pandemic period in Mexico, Colombia, and Ecuador by conducting an empirical study in the selected universes. However, the research has limitations. The study was only from perspective quantitatively, so it is necessary to investigate the qualitative approach to know consumers' feelings, emotions, and explanatory reasons in their purchasing behavior. Moreover, with the results obtained, it can be inferred that it happens in all generational cohorts (BabyBoomer, Gen X, Gen, and Gen Z), regardless of gender (male or female), all behave the same way, in times of pandemic. However, this study did not intend to measure the frequency of purchase as a variable to measure online purchasing behavior due to the pandemic period: online and at home. The results found and analyzed offer information on the intention to buy online that ad hoc to the world's current situation due to the health contingency caused by Covid-19. It provides knowledge on the behavior of purchase and consumption of consumers in Latin American countries.

Adding direct-purchase intention elements and exploring the relationship between behavior would provide more decisive conclusions for this assumption. However, one of the research questions about the intention to buy vegetables and fruits in virtual stores revealed the intention of Latin American consumers to buy these products, resulting in the fact that they prefer to buy fresh products from first necessity in traditional markets, despite the health contingency. Data, which can contribute to business knowledge to MSMEs in the sample countries. On average, 97% of companies are Mipyme (neighborhood, convenience, and family retail stores). Nevertheless, these companies must learn to adapt the commercial messages and the products and services to the target culture. It is a sine qua non condition to reach the consumer and ensure the first step towards commercial success. Besides, we must consider that the Latin American consumer does not have experience (compared to Europeans or North Americans) in online consumption and needs the necessary tools to make electronic purchases. This situation can be an opportunity or cause of failure for the company if it is not treated correctly, especially in social isolation and restriction on coexistence.

Experts in economics, finance, and business mention that many business sectors will be dead for many years due to the pandemic. The tourism, aeronautics, the automotive, and the entertainment sector (among others more classified as non-essential) are zombies business (they are dead trying to survive in a world of the living). When plotting the economic outlook for the coming years, if the behavior of economic recovery would be graphed, there would be a fall and eventual recovery, a graph in the form of a chair would be had. That is to say, stagnation and, then, further down the fall, the world economy will fall, then it will stabilize for a time, and then it will fall again. With this situation, in research like this, it can be seen that companies belonging to the economy of the life sector will flourish in the coming years. Food companies (retailing and agriculture), health (well-being and hygiene) will do very well.

With this research, we can see that companies belonging to the life sector's economy will flourish in the coming years. Food (retailing and agriculture), health (well-being and hygiene), biotechnology, education, telecommunications, technology (virtual and augmented reality), and especially digital markets. They are companies that will have successful and survive in the pandemic period.

The trend of distance consumption will allow more than 2.5 billion people to work remotely, overnight. It was already known in recent years that teleworking would occur. However, humanity did not expect that it would be so fast and under pressure in the face of a pandemic like Covid-19, although humanity, from her different perspectives (citizen, consumer, student, medical, or housekeeper), understand under pressure, can change very Quick. Also, humanity learned fairly quickly that this is a global event, not something local and that a problem in one place is a problem everywhere. So individualism, in terms of countries, the tendency to isolation can generate business opportunities for small businesses.

## Declarations

### Author contribution statement

Emigdio Larios-Gómez: Conceived and designed the experiments; Performed the experiments; Analyzed and interpreted the data; Contributed reagents, materials, analysis tools or data; Wrote the paper.

Laura Fischer: Contributed reagents, materials, analysis tools or data; Wrote the paper.

Mónica Peñalosa: Performed the experiments; Analyzed and interpreted the data.

Mayra Ortega: Performed the experiments.

### Funding statement

This research did not receive any specific grant from funding agencies in the public, commercial, or not-for-profit sectors.

### Data availability statement

Data included in article/supplementary material/referenced in article.

### Declaration of interests statement

The authors declare no conflict of interest.

### Additional information

No additional information is available for this paper.

## Appendix A Supplementary data

REPORTE COMPLETO V2.0 COVID 19MX CO EC
